# Human Cytomegalovirus Latency and Reactivation in Allogeneic Hematopoietic Stem Cell Transplant Recipients

**DOI:** 10.3389/fmicb.2019.01186

**Published:** 2019-05-28

**Authors:** Lauren Stern, Barbara Withers, Selmir Avdic, David Gottlieb, Allison Abendroth, Emily Blyth, Barry Slobedman

**Affiliations:** ^1^Discipline of Infectious Diseases and Immunology, Sydney Medical School, Charles Perkins Centre, University of Sydney, Sydney, NSW, Australia; ^2^Department of Haematology, St Vincent’s Hospital, Darlinghurst, NSW, Australia; ^3^Westmead Institute of Medical Research, University of Sydney, Sydney, NSW, Australia; ^4^Sydney Cellular Therapies Laboratory, Westmead, NSW, Australia; ^5^Blood and Marrow Transplant Unit, Westmead Hospital, Sydney, NSW, Australia

**Keywords:** human cytomegalovirus, HCMV, CMV, latency, reactivation, hematopoietic stem cell transplant, HSCT

## Abstract

Human cytomegalovirus (HCMV) reactivation is a major infectious cause of morbidity and mortality after allogeneic hematopoietic stem cell transplantation (HSCT). HCMV is a ubiquitous beta-herpesvirus which asymptomatically infects immunocompetent individuals but establishes lifelong latency, with the potential to reactivate to a life-threatening productive infection when the host immune system is suppressed or compromised. Opportunistic HCMV reactivation is the most common viral complication following engraftment after HSCT and is associated with a marked increase in non-relapse mortality, which appears to be linked to complex effects on post-transplant immune recovery. This minireview explores the cellular sites of HCMV latency and reactivation in HSCT recipients and provides an overview of the risk factors for HCMV reactivation post-HSCT. The impact of HCMV in shaping post-transplant immune reconstitution and its relationship with patient outcomes such as relapse and graft-versus-host disease will be discussed. Finally, we survey current and emerging strategies to prevent and control HCMV reactivation in HSCT recipients, with recent developments including adoptive T cell therapies to accelerate HCMV-specific T cell reconstitution and new anti-HCMV drug therapy for HCMV reactivation after HSCT.

## Introduction

Human cytomegalovirus (HCMV) is a beta-herpesvirus carried by a majority of the global population (Cannon et al., 2010; Zuhair et al., 2019). The seroprevalence of HCMV increases with age and has an estimated global mean of 83% (Zuhair et al., 2019). While primary infection is usually asymptomatic in immunocompetent individuals, the virus establishes a lifelong latent infection that is never eliminated by the host immune system. Intermittent subclinical viral reactivation events are thought to be controlled by effective immune surveillance and may drive the high frequencies of HCMV-specific T-cells found in the peripheral blood of healthy seropositive individuals (Sester et al., 2002; Sylwester et al., 2005). By contrast, reactivation from latency is responsible for significant morbidity and mortality in immunocompromised and immunosuppressed populations,including solid-organ transplant and allogeneic hematopoietic stem cell transplant (HSCT) recipients, HIV/AIDS patients and the developing fetus.

HCMV reactivation is the major viral infectious complication after allogeneic HSCT and is associated with an increased risk of non-relapse mortality (Takenaka et al., 2015; Green et al., 2016; Teira et al., 2016), which is not principally attributable to direct HCMV disease (Green et al., 2016). Uncontrolled HCMV replication following reactivation can lead to life-threatening end-organ disease, commonly manifesting as pneumonia and gastrointestinal disease, and less frequently as retinitis, hepatitis and encephalitis. Reactivation may also indirectly contribute to detrimental patient outcomes through antiviral drug toxicities and complex impacts on post-transplant immune reconstitution, including links with graft-versus-host disease (GvHD) (Cantoni et al., 2010) and microbial superinfections (Nichols et al., 2002; Yong et al., 2017a). There is no licensed HCMV vaccine and current antiviral agents are limited by their toxic side-effects and the risk of antiviral drug resistance.

The cellular sites and mechanisms associated with HCMV latency and reactivation are incompletely understood, in part due to the high human-specificity of HCMV which precludes the use of animal models to directly study HCMV infection. Clinical evidence indicates that the risk of reactivation post-HSCT is strongly connected to the pre-transplant HCMV serostatus of the donor and recipient (Boeckh and Nichols, 2004; George et al., 2010) and the pace of post-transplant HCMV-specific T-cell recovery. However, the lack of well-validated biomarkers for reactivation makes it challenging to predict the incidence and outcome of infection in individual patients, and greater knowledge of the influence of HCMV on post-transplant immune reconstitution is required.

## HCMV Reactivation in Allogeneic Hematopoietic Stem Cell Transplantation (HSCT)

Allogeneic HSCT is the only curative option for many hematological malignancies and diseases (Copelan, 2006). GvHD, opportunistic infections and relapse are the leading causes of mortality in the first 2 years post-transplant (Gratwohl et al., 2005; D’Souza and Fretham, 2018). The reactivation of latent double-stranded DNA viruses is common in the early post-engraftment period (Hill et al., 2017) and HCMV reactivation is the most frequent opportunistic viral infection after HSCT. Virological surveillance for HCMV replication is routinely performed in the first 100 days post-transplant through quantitative PCR monitoring for HCMV genomes in blood plasma (Emery et al., 2000), with HCMV DNAemia being an independent risk factor for disease (Zaia et al., 1997) and non-relapse mortality (Hiwarkar et al., 2013; Green et al., 2016; Ramanathan et al., 2016; Teira et al., 2016).

Reactivation develops in over 60% of HCMV seropositive recipients (R+), and in approximately 10% of seronegative recipients (R-) transplanted from seropositive donors (D+) (George et al., 2010). In R+/D+ patients, reactivation may derive from endogenous latent HCMV in the seropositive recipient (Winston et al., 1985; Kawasaki et al., 1999) and/or from latently infected cells transferred within the seropositive donor stem cell allograft. Recipient seropositivity alone is an adverse prognostic factor for overall survival (Broers et al., 2000; Craddock et al., 2001). Seropositive recipients who receive grafts from HCMV-naïve donors (R+/D-) experience the highest incidence of HCMV reactivation and disease (Ljungman et al., 2006; Webb et al., 2018), a likely consequence of delayed HCMV-specific immune recovery owing to the lack of pre-existing HCMV-specific memory lymphocytes in the graft (Cwynarski et al., 2001; Zhou et al., 2009). As the recipient’s existing cellular immune system is eradicated by transplant conditioning regimens, R+/D- patients rely on *de novo* T-cell reconstitution via the thymus from donated pluripotent hematopoietic stem cells to generate a primary T-cell response to HCMV reactivation. By contrast, HCMV-specific memory T-cells contained in D+ grafts can undergo more rapid antigen-driven expansion within the recipient in the early post-transplant period and contribute to early control of reactivation (Cwynarski et al., 2001; Gandhi et al., 2003; Scheinberg et al., 2009). For a seropositive recipient, the choice of a seropositive over seronegative donor offers a survival advantage in the setting of myeloablative unrelated donor transplantation, but the effect on survival with matched sibling donors is less strong (Ljungman et al., 2003, 2014). Nonetheless, due to the high incidence of HCMV-related complications in R+/D- patients, where possible, attempts are made to match serostatus in donors and recipients.

Advances in transplant and antiviral treatment practices over the last 25 years have reduced the incidence of HCMV disease to ∼10% in the first year post-transplant (Boeckh and Ljungman, 2009; Green et al., 2016). However, the use of prophylactic or pre-emptive antiviral therapy delays HCMV-specific T-cell recovery (Li et al., 1994) and has led to increasing rates of late HCMV reactivation and disease (Einsele et al., 2000). Additionally, HCMV pneumonia remains associated with high (up to 70%) mortality (Erard et al., 2015). Gastrointestinal HCMV disease often develops without detection of HCMV DNAemia (Cho et al., 2013; Gabanti et al., 2015) and can be difficult to distinguish from gastrointestinal GvHD, often occurring in the same patients (Cho et al., 2013; Bhutani et al., 2015).

## Cellular Sites of HCMV Latency and Reactivation

The transition from viral latency to reactivation underpins the pathogenesis of HCMV in HSCT. HCMV latency is characterized by maintenance of the viral genome as an intranuclear episome (Bolovan-Fritts et al., 1999) without replication, but with the potential to reactivate to a productive infection. A wide range of cell types support productive infection (Ibanez et al., 1991; Sinzger et al., 2008), but latency appears to be restricted to primitive bone-marrow-resident CD34^+^ cells and CD33^+^ myeloid progenitor cells (Mendelson et al., 1996; Hahn et al., 1998; Reeves et al., 2005b), which retain the latent viral genome as they differentiate into peripheral blood CD14^+^ monocytes and myeloid dendritic cells (mDCs) (Taylor-Wiedeman et al., 1991, 1994; Hahn et al., 1998; Khaiboullina et al., 2004; Reeves et al., 2005b). A recent study found that CD14^+^ monocytes expressing the surface marker B7-H4 were a predominant site of latency in peripheral blood of healthy donors (Zhu et al., 2018). It may be that HCMV preferentially infects early myeloid progenitors or promotes the differentiation of infected pluripotent CD34^+^ cells to myeloid-lineage subsets that support latency (Zhu et al., 2018).

Latently infected cells contain HCMV DNA (Minton et al., 1994) but do not support infectious virus production. The terminal differentiation to mature mDCs and macrophages is accompanied by chromatin remodeling of the HCMV major immediate-early promoter (Reeves et al., 2005a,b), which facilitates reactivation of the lytic gene cascade and the production of infectious virus (Taylor-Wiedeman et al., 1994; Reeves et al., 2005b; Reeves and Sinclair, 2013; Poole et al., 2015). Allogeneic stimulation (Soderberg-Naucler et al., 1997) and pro-inflammatory cytokines such as IFN-γ, TNF, and IL-6 are implicated in driving myeloid cell maturation and reactivation (Fietze et al., 1994; Söderberg-Nauclér et al., 2001; Hargett and Shenk, 2010; Reeves and Compton, 2011; Huang et al., 2012; Reeves and Sinclair, 2013; Forte et al., 2018).

Latently infected cells are present at very low frequencies (0.004–0.01% of mononuclear cells) in GCSF-mobilized peripheral blood or bone-marrow from healthy seropositive donors (Slobedman and Mocarski, 1999), but underlie the capacity for iatrogenic transmission of latent HCMV through D+ HSCT allografts. Additionally, the high risk of reactivation in R+/D- patients suggests that pre-transplant conditioning regimens incompletely eradicate latent HCMV reservoirs in the recipient (Wills et al., 2015). It also remains possible there are additional sites of HCMV latency, with conflicting evidence regarding possible latency in aortic endothelial cells (Fish et al., 1998; Pampou et al., 2000; Reeves et al., 2004). Whether HCMV establishes a low-level productive infection in bone-marrow stromal cells (Taichman et al., 1997; Smirnov et al., 2007; Soland et al., 2014) or in a self-renewing CD34^+^ cell subset (Goodrum et al., 2004) also remains unclear, yet HCMV DNA has been detected in diverse tissue sites (Hendrix et al., 1997; Chen and Hudnall, 2006; Gordon et al., 2017) and recent RNA-seq uncovered HCMV transcripts at multiple locations, including the ovaries, blood, adipose tissue, and lung (Shnayder et al., 2018). The specific cell types harboring HCMV in these studies and whether they represent productive, abortive, or latent infection is unknown. The widespread prevalence of HCMV within asymptomatic individuals nonetheless highlights the importance of host immune control in preventing unchecked HCMV replication leading to life-threatening disease.

## Risk Factors for HCMV Reactivation After HSCT

In addition to recipient and donor HCMV serostatus, independent risk factors for reactivation include increasing recipient age (Tong et al., 2013; Takenaka et al., 2015), use of unrelated or HLA-mismatched donors (Qayed et al., 2014; Takenaka et al., 2015), T-cell depletion (Walker et al., 2007; Yoon et al., 2009), GvHD (Walker et al., 2007; George et al., 2010; Qayed et al., 2014; Cohen et al., 2015), and high-dose corticosteroids for GvHD (Yanada et al., 2003; Tong et al., 2013; Melendez-Munoz et al., 2019). T-cell depletion and prolonged steroid therapy mitigate GvHD but delay antiviral T-cell reconstitution (Aubert et al., 2001; Craddock et al., 2001; Wagner et al., 2005; Lilleri et al., 2009; Tormo et al., 2011). A high incidence of HCMV reactivation is also observed after T-cell replete haploidentical HSCT with post-transplant cyclophosphamide (Di Stasi et al., 2014; Crocchiolo et al., 2015; Goldsmith et al., 2016; Slade et al., 2017).

The reconstitution kinetics of HCMV-specific T-cells post-HSCT have a close relationship with the risk and prognosis of reactivation (Lilleri et al., 2006; Gratama et al., 2010; Espigado et al., 2014). HCMV-specific CD4^+^ and CD8^+^ T-cells expand with reactivation and are likely both required for control and/or protection (Foster et al., 2002; Sacre et al., 2008; Widmann et al., 2008; Pourgheysari et al., 2009; Lilleri et al., 2012; Gabanti et al., 2015; Raeiszadeh et al., 2015; Ciaurriz et al., 2017). Quantitative thresholds of CD8^+^ and CD4^+^ HCMV-specific T-cells associated with protection from, or control of, reactivation or disease post-HSCT have been defined using HLA tetramers or *ex vivo* viral stimulation assays (Aubert et al., 2001; Cwynarski et al., 2001; Gratama et al., 2001, 2010; Lilleri et al., 2008; Moins-Teisserenc et al., 2008; Borchers et al., 2011, 2012; Tormo et al., 2011; Lilleri et al., 2012; Liu et al., 2016). Threshold numbers have not been well-validated for routine clinical use and are less informative of protection from reactivation and disease in patients under steroid therapy or with prior GvHD (Lilleri et al., 2012; Gabanti et al., 2015).

Control of reactivation may depend more heavily on the functional recovery of HCMV-specific T-cell immunity (Quinnan et al., 1982; Reusser et al., 1991; Ozdemir et al., 2002; Nakamura et al., 2004; Gratama et al., 2008; Zhou et al., 2009; Tormo et al., 2010; Krol et al., 2011; Tey et al., 2013; Espigado et al., 2014; Ciaurriz et al., 2017). Polyfunctional HCMV-specific T-cell responses post-HSCT are associated with lower viral loads, protection from subsequent episodes of reactivation and lower antiviral therapy requirements (Zhou et al., 2009; Munoz-Cobo et al., 2012; Gimenez et al., 2015; Pelak et al., 2017). Delayed or undetectable HCMV-specific cytotoxic T-cell responses are prominent risk factors for HCMV disease (Reusser et al., 1991; Gratama et al., 2001; Ganepola et al., 2007). Camargo et al. (2019) recently described a composite biomarker comprising a protective (IL-2+IFN-γ+TNF-α+MIP-1β+) and non-protective (IL-2-IFN-γ+TNF-α-MIP-1β+) CD8^+^ T-cell cytokine signature in response to *in vitro* HCMV pp65 peptide stimulation that independently predicted the risk of clinically significant reactivation. Assessment of HCMV-specific immunity through measurement of whole blood *ex vivo* IFN-γ secretion responses to HCMV peptides has also emerged as a promising prognostic approach for HCMV reactivation post-HSCT (Tey et al., 2013; Yong et al., 2017b). However, it is argued that the selective recovery of HCMV-specific T-cell immunity ahead of global T-cell reconstitution carries a higher risk of subsequent reactivation (Tey et al., 2014).

The detection of reactivation prior to 100 days post-transplant (Boeckh et al., 2003; Kim et al., 2004; Liu et al., 2015), plasma viral load (Zaia et al., 1997), leukopenia (Jang et al., 2012), lymphopenia (Einsele et al., 1993), and GvHD (Boeckh et al., 2003; Ljungman et al., 2006; Green et al., 2012) represent additional risk factors for HCMV disease. Donor grafts with ≥5 activating killer-cell immunoglobulin-like receptor (KIR) genes or with both KIR2DS2 and KIR2DS4 predict a lower risk of reactivation (Zaia et al., 2009), and the use of donors with multiple or additional activating KIRs is associated with a lower incidence of reactivation (Chen et al., 2006; Cook et al., 2006).

## Impact of HCMV Reactivation on Post-HSCT Immune Recovery, Relapse and GvHD

The immune system crucially regulates the risk of HCMV reactivation and disease, but HCMV itself also has a profound influence in shaping immune profiles in healthy seropositive individuals (Chidrawar et al., 2009; Brodin et al., 2015; Patin et al., 2018) and HSCT patients (Itzykson et al., 2015; Lakshmikanth et al., 2017), although the functional implications of this immune modulation are not yet clear. In some HSCT recipients, reactivation might be an epiphenomenon of poor immune reconstitution, but it is also possible that the immunomodulatory effects of HCMV infection may contribute to poor post-transplant outcomes (Nichols et al., 2002). Indeed, while HCMV DNAemia-guided pre-emptive antiviral therapy has reduced the incidence of HCMV disease post-HSCT, the survival disadvantage associated with HCMV infection has not been eliminated (Broers et al., 2000; Schmidt-Hieber et al., 2013; Green et al., 2016). HCMV encodes a range of immunomodulatory gene products that are expressed during both productive infection and latency, including a homolog of the immunosuppressive cytokine IL-10 (Jenkins et al., 2004; McSharry et al., 2012; Avdic et al., 2014; Young et al., 2017). HCMV infection in HSCT recipients increases the risk of bacterial and fungal superinfections (Nichols et al., 2002; Yong et al., 2017a) and GvHD (Lonnqvist et al., 1984; Broers et al., 2000; Cantoni et al., 2010), which might be connected to complex impacts of HCMV reactivation and/or its treatment on post-transplant immune recovery.

### T Cell Reconstitution

Reactivation stimulates the recovery of HCMV-specific T-cells after HSCT (see section “Risk Factors for HCMV Reactivation After HSCT”) (Hakki et al., 2003; Tormo et al., 2010; Ciaurriz et al., 2017), but is also accompanied by broader changes in the T-cell compartment. Patients with reactivation display accelerated CD8^+^ T-cell reconstitution in the first year post-transplant (Lugthart et al., 2014; Drylewicz et al., 2016), which is likely to be driven by clonal expansions of HCMV-specific effector-memory αβ CD8^+^ T-cells (Suessmuth et al., 2015; Link et al., 2016), leading to an inverted CD4:CD8 ratio. Deep sequencing of the TCR-β repertoire at 1 year post-HSCT uncovered a contraction in effector-memory CD8^+^ TCR diversity and holes in the underlying effector-memory CD8^+^ T-cell repertoire in patients who had experienced reactivation (Suessmuth et al., 2015). The selective expansion of HCMV-reactive Vδ2^neg^ γδ T-cells (Knight et al., 2010; Scheper et al., 2013), and clonal (Vγ9^neg^ and Vδ2^neg^) γδ T-cell proliferations suggestive of adaptive responses (Ravens et al., 2017), are also observed following reactivation. Reactivation also triggers the expansion of large granular lymphocytes (Nann-Rütti et al., 2012). Lugthart et al. (2014) found the reconstitution of naïve and central memory T-cells up to 2 years post-transplant was not compromised by reactivation, but Suessmuth et al. (2015) observed lower numbers of naïve T-cells in the first year post-HSCT in patients with reactivation. Many of the immunological features associated with HCMV reactivation after HSCT, including oligoclonal expansions of terminally differentiated HCMV-specific T-cells, are also found with aging in seropositive individuals (Khan et al., 2002; Hadrup et al., 2006), although recently it was reported that these HCMV-induced clonal T-cell expansions may not compromise CD8^+^ T-cell repertoire diversity in the elderly (Lindau et al., 2019). The prominent influence of HCMV seropositivity and reactivation in shaping global immune reconstitution signatures after HSCT is apparent (Itzykson et al., 2015), but the impact of reactivation on immune recovery beyond 2 years post-transplant is not well-characterized.

### NK Cell Reconstitution

HCMV reactivation drives a rapid expansion of IFN-γ-producing NKG2C^+^ NK cells (Foley et al., 2012b), which likely contribute to early control of reactivation (Kheav et al., 2014; Davis et al., 2015; Muccio et al., 2016). Expanded proportions of mature (CD56^dim^CD57^+^NKG2A^-^CD158b^+^) NKG2C^+^ NK cells persist after viral clearance (Foley et al., 2012b) and memory-like expansions of NKG2C^+^CD57^+^ NK cells are also observed in R+/D+ patients with subclinical HCMV infection (Foley et al., 2012a). Specific recognition by NKG2C^+^ NK cells of HCMV UL40 peptides presented in the context of HLA-E was recently identified to be the mechanism that drives the expansion and differentiation of NKG2C^+^ NK cells during HCMV infection (Hammer et al., 2018).

### Relapse

An association between early HCMV reactivation and reduced myeloid leukemia relapse has been reported (Lonnqvist et al., 1986; Elmaagacli et al., 2011; Green et al., 2013; Ito et al., 2013; Takenaka et al., 2015; Peric et al., 2018). This putative protective effect might be mediated through the anti-leukemic activities of CD56^dim^CD57^+^NKG2C^+^ NK cell and Vδ2^neg^ γδ T-cell subsets which expand with reactivation (Scheper et al., 2013; Cichocki et al., 2016), or via enhancement of donor alloimmune responses in the presence of infection and HCMV-specific CD8^+^ T-cells (Ogonek et al., 2017; Varanasi et al., 2019). However, the role of reactivation in protection from malignancy relapse post-HSCT is controversial, as others have not found evidence of this association in patients with acute myeloid leukemia, chronic myeloid leukemia, acute lymphoid leukemia, lymphoma, or myelodysplastic syndrome (Nakamura et al., 2004; Green et al., 2013; Jeljeli et al., 2014; Mariotti et al., 2014; Takenaka et al., 2015; Teira et al., 2016; Ramanathan et al., 2016; Admiraal et al., 2017). Further studies are thus required to better define the patient subgroups and immunological features associated with possible relapse protection in HSCT patients with HCMV reactivation.

### Graft-Versus-Host Disease

Graft-versus-host disease and its steroid therapy increase the risk of reactivation after HSCT (Miller et al., 1986; Yanada et al., 2003; Ljungman et al., 2006; George et al., 2012; Liu et al., 2015; Valadkhani et al., 2016). The alloimmune responses mediating GvHD impair thymopoiesis (Weinberg et al., 2001; Castermans et al., 2011) and delay HCMV-specific T-cell reconstitution, and high-dose steroids impair the recovery of HCMV-specific T-cells in a dose-dependent manner (Hakki et al., 2003; Widmann et al., 2008). Further, it has been speculated that the proinflammatory immune environment associated with GvHD may promote reactivation, as has been demonstrated following allogeneic stimulation of latently infected cells *ex vivo* (Soderberg-Naucler et al., 1997). Conversely, patients with reactivation more frequently develop GvHD (Lonnqvist et al., 1984; Janeczko et al., 2016) and the risk of extensive chronic GvHD was reduced with the use of HCMV DNAemia-guided pre-emptive antiviral therapy (Larsson et al., 2004). An increased risk of acute GvHD was observed during episodes of active HCMV replication after HSCT, supporting a bidirectional relationship between reactivation and GvHD (Cantoni et al., 2010). Further research is required to delineate the mechanisms underlying this phenomenon, but an inflammatory response to reactivation or the potential cross-reactivity of HCMV-specific T-cells with human alloantigens (Hall et al., 2017) might play a role.

## Prevention and Treatment Strategies

Standard antiviral drugs for reactivation after HSCT are ganciclovir, valganciclovir and foscarnet. Prophylactic use is reserved for high-risk patients due to their significant toxic side effects. Ganciclovir causes neutropenia which increases the risk of bacterial and fungal superinfections (Goodrich et al., 1993; Boeckh et al., 1996; Broers et al., 2000; Einsele et al., 2000; Yanada et al., 2003), while foscarnet and cidofovir (used as a second- or third- line therapy) (Ljungman et al., 2008) are nephrotoxic (Ljungman et al., 2001; Reusser et al., 2002). Additional concerns relate to the development of antiviral drug resistance (Campos et al., 2016; Servais et al., 2016; Chemaly et al., 2018) and breakthrough reactivation or disease (Green et al., 2012). HCMV drug resistance has been reported in up to 14.5% of HSCT recipients receiving pre-emptive therapy (Shmueli et al., 2014). Prolonged antiviral exposure (Boeckh and Ljungman, 2009), suboptimal prophylactic dosing (Lischka et al., 2016), corticosteroid therapy (Frietsch et al., 2019) and delayed immune reconstitution foster the selection of drug resistant HCMV mutants. Mutations in the HCMV UL97 (viral protein kinase) gene confer resistance to (val)ganciclovir and maribavir (Marfori et al., 2007; Piret and Boivin, 2019), while HCMV UL54 (viral DNA polymerase) gene mutations are associated with resistance to foscarnet, cidofovir and (val)ganciclovir (El Chaer et al., 2016). Infection with multiple HCMV genotypes is associated with reduced efficacy of antiviral treatment (Zawilinska et al., 2016; Vinuesa et al., 2017). Importantly, current drugs do not target HCMV during latency, as these drugs target the viral replication machinery, and latency is typified by maintenance of the viral genome without replication. This highlights the potential for clinically relevant recurrence of reactivation following therapy cessation.

Prophylactic administration of Letermovir, a new anti-HCMV agent which inhibits the viral terminase complex, recently demonstrated the capacity to reduce the risk of HCMV disease and all-cause mortality at 24 weeks post-transplant, in a Phase 3 trial (Marty et al., 2017). There are, however, reports emerging of breakthrough viraemia and disease associated with HCMV UL56 terminase mutations conferring Letermovir resistance in HSCT recipients (Lischka et al., 2016; Knoll et al., 2018; Frietsch et al., 2019). Letermovir has now been licensed for HCMV prophylaxis after HSCT and its efficacy in treating refractory or resistant HCMV infection and disease will be evaluated in an upcoming Phase 2 trial (NCT03728426). Given its unique mechanism of action, combination therapy of Letermovir with other currently licensed antivirals (Wildum et al., 2015) may represent a means to more effectively control HCMV and limit the emergence of antiviral drug resistance in HSCT patients, although this area remains to be explored.

Pre-emptive treatment based on viral DNAemia surveillance (Emery et al., 2000) minimizes toxic drug exposure and reduces rates of HCMV disease and mortality (Einsele et al., 1995; Ljungman et al., 1998; Reusser et al., 2002), but there is no consensus on the appropriate plasma viral load threshold for initiating such therapy (Green et al., 2012; Tan et al., 2015; Green et al., 2016; Hanna et al., 2017). Inter-laboratory assays for HCMV DNA quantitation vary and the WHO reference standard for HCMV DNA lacks commutability in many assays (Hayden et al., 2015). Lower viral load thresholds may be required in settings of corticosteroid treatment and T-cell depletion (Green et al., 2012; Melendez-Munoz et al., 2019). Monitoring both HCMV-specific T-cell immunity and viral load has recently been successfully applied to guide the withholding or early discontinuation of antiviral treatment (Avetisyan et al., 2007; Navarro et al., 2016; Kumar et al., 2017). Further to characterizing immune reconstitution profiles in patients who spontaneously resolve reactivation without antiviral treatment (Camargo et al., 2019), more detailed investigation of the immune environment prior to the detection of HCMV DNAemia should be a focus of future studies to optimize the identification of high-risk patients and timing of pre-emptive therapy.

Pooled HCMV-specific or polyclonal intravenous immunoglobulin is not effective at preventing reactivation or reducing mortality when used in the treatment of HCMV pneumonia post-HSCT (Raanani et al., 2009; Erard et al., 2015), although strain-specific antibody therapy was recently shown to potently inhibit murine cytomegalovirus (MCMV) reactivation after bone-marrow transplantation in a preclinical murine model (Martins et al., 2019). Adoptive HCMV-specific T-cell therapies to prevent and treat reactivation after HSCT have been developed since the early 1990s (Riddell et al., 1992; Walter et al., 1995; Mackinnon et al., 2008). Third-party- or stem cell donor-derived HCMV-specific T-cells expanded *ex vivo* or isolated directly with HCMV-specific tetramers and infused in the post-transplant period can accelerate HCMV-specific immune recovery and contribute to long-term control of reactivation and protection from HCMV disease (Peggs et al., 2009; Peggs et al., 2011; Neuenhahn et al., 2017; Withers et al., 2017). The post-transplant infusion of donor-derived HCMV-specific cytotoxic T-cells was shown to reduce the requirement for antiviral drug therapy in a Phase 2 trial (Blyth et al., 2013). Further studies are needed to determine the optimal timing of adoptive cell infusion and understand its impact on post-transplant immune reconstitution.

## Conclusion

HCMV is a highly prevalent, opportunistic pathogen that continues to cause substantial morbidity and mortality after HSCT. Improved knowledge of the cellular sites of HCMV latency and the conditions which enable its reactivation to clinically significant infection will be needed to better predict, prevent and control reactivation post-HSCT. Future strategies might involve the selective depletion of latently infected cells from the graft (Krishna et al., 2016, 2017), plasma metabolomics profiling to predict the emergence of reactivation (Monleon et al., 2018), the vaccination of transplant recipients and donors to enhance HCMV-specific immune reconstitution (Kharfan-Dabaja et al., 2012; Ma et al., 2018), or the engineering of corticosteroid-resistant HCMV-specific T-cells to improve adoptive cell therapies (Menger et al., 2015). The marked impact of HCMV on post-transplant immune reconstitution warrants continued research to understand its relationship with patient outcomes. New therapeutic approaches for reactivation are actively being pursued and it is hoped these will lessen the clinical impact of reactivation after HSCT in the near future.

## Author Contributions

LS and BW generated the initial draft of the manuscript. All authors contributed to the subsequent writing and review of the manuscript.

## Conflict of Interest Statement

The authors declare that the research was conducted in the absence of any commercial or financial relationships that could be construed as a potential conflict of interest.
